# Level of Immersion in Virtual Environments Impacts the Ability to Assess and Teach Social Skills in Autism Spectrum Disorder

**DOI:** 10.1089/cyber.2014.0682

**Published:** 2016-04-01

**Authors:** Haylie L. Miller, Nicoleta L. Bugnariu

**Affiliations:** Department of Physical Therapy, University of North Texas Health Science Center, Fort Worth, Texas.

## Abstract

Virtual environments (VEs) may be useful for delivering social skills interventions to individuals with autism spectrum disorder (ASD). Immersive VEs provide opportunities for individuals with ASD to learn and practice skills in a controlled replicable setting. However, not all VEs are delivered using the same technology, and the level of immersion differs across settings. We group studies into low-, moderate-, and high-immersion categories by examining five aspects of immersion. In doing so, we draw conclusions regarding the influence of this technical manipulation on the efficacy of VEs as a tool for assessing and teaching social skills. We also highlight ways in which future studies can advance our understanding of how manipulating aspects of immersion may impact intervention success.

## Introduction

Virtual social skills interventions, in which a therapist and patient control avatars and interact in a virtual environment (VE), have received recent attention. This new approach may provide a means of delivering services to patient populations with limited access to care, for example, in rural communities. The increasing prevalence of smartphones, tablets, and gaming systems presents an opportunity to deliver services on varied platforms and in highly interactive ways.^1^ Interest in technology- or computer-based intervention for autism spectrum disorder (ASD) emerged over 40 years ago^2^ and continues today, in part, because parents and educators of children with ASD report more positive responses to digital media than humans. However, the benefits of virtual versus face-to-face intervention remain unclear.

Researchers have explored the application of VEs to neuroscience and the assessment and treatment of adult and pediatric clinical populations.^3–5^ VEs and other computer-administered tasks offer opportunities to learn and practice skills in a controlled repeatable setting with less social pressure than face-to-face interaction.^6^ However, the implementation of this technology remains widely variable.

Several reviews and meta-analyses have examined the use of computer technologies and VEs in assessment and treatment of the social symptoms of ASD.^7–14^ Some focused on ability to control the environment,^9,11^ some included a broad range of technologies (e.g., computer games, robots),^14^ and others adopted the perspective of cognitive theories.^12^ To our knowledge, at the time of this review, Grynszpan et al.^10^ conducted the only related meta-analysis, finding evidence that technology-based interventions are effective in teaching social skills to individuals with ASD, despite limited availability of randomized controlled trials.

Many reviews note that the literature is still limited by high variability in sample characteristics and methodology, which impede cross-study comparisons. Although some referenced the potential impact of level of immersion and the sense of presence generated by VEs,^11^ few systematically explored the effect of level of immersion. Many reviews included all computer-mediated interventions, regardless of whether they used a VE. For the purpose of this discussion, we will adhere to the traditional definition of a VE: an interactive computer environment that gives the illusion of *displacement to a different location.*^15^

In this study, we present peer-reviewed articles that use traditionally defined VEs as a platform to assess and teach social skills in ASD. We critically evaluate the level of immersion used in previous studies (low, moderate, and high immersion; Table 1). We also highlight unanswered questions about the generalizability of skills learned in the virtual world, the level of immersion required to successfully teach social skills, and the variability in treatment response across individuals with ASD with differing symptom profiles. In doing so, we aim to propose a possible theoretical framework for examining the level of immersion used in a study. However, it is important to note that this approach must be empirically tested in future work to determine whether it is (a) a valid means of quantifying immersion and (b) a useful approach to evaluating whether a given VE will be appropriate for use in assessing or treating social skills in ASD.

**Table 1.** Examples of Virtual Environment Characteristics by Level and Aspect of Immersion

	*Aspect of immersion*				
*Level of immersion*	*Inclusive*	*Extensive*	*Surrounding*	*Vivid*	*Matching*
Low	Numerous signals indicating the presence of device(s) in the physical world (e.g., use of a joystick or mouse to control the VE, direct instruction from an experimenter during the task)	Only accommodates 1 sensory modality (e.g., auditory, visual, motor/proprioceptive); stimuli are not spatially oriented	Computer monitor presentation with limited field of view	Low fidelity and visual/color resolution; display may replicate features of the simulated environment, but not in a detailed or specific manner	No motion capture; visual experience does not match proprioceptive feedback
Moderate	Some signals indicating the presence of device(s) in the physical world (e.g., noise from a computer fan, weight and movement restriction from wearing a safety harness)	Accommodates 1–2 sensory modalities (e.g., auditory, visual, motor/proprioceptive); stimuli may or may not be spatially oriented	Large-screen projection with extended field of view	Moderate fidelity and visual/color resolution; display replicates some features of the simulated environment, but some detail may be missing	Body segment motion capture (e.g., head, hand); visual experience somewhat altered to match proprioceptive feedback based on head or body segment movement
High	Limited signals indicating the presence of device(s) in the physical world (e.g., the weight of an HMD or an eye-tracking device)	Accommodates >2 sensory modalities (e.g., auditory, visual, motor/proprioceptive); stimuli are spatially oriented	Head-mounted device or surround projection	High fidelity and visual/color resolution; display closely replicates multiple features of the simulated environment in great detail (e.g., correctly placed, dynamic shadows)	Full-body motion capture; visual experience altered to closely match proprioceptive feedback based on whole body movement

HMD, head mounted device; VE, Virtual environment.

## Interplay Between Level of Immersion and Sense of Presence

### Level of immersion

VEs can be presented in various ways, from computer interfaces that resemble traditional video games to systems involving motion capture and full-body interaction with virtual objects. The level of immersion is a technical manipulation that can be applied to a broad range of paradigms. Slater and Wilbur^16^ proposed that the immersive capability of a VE depends on the degree to which it is *inclusive*, *extensive*, *surrounding*, *vivid*, and *matching*. Each aspect influences, but is not the sole determinant of, the user's perceptual experience.

*Inclusive* refers to whether a VE eliminates signals indicating the existence of a physical world separate from the virtual world (e.g., joystick, weight of wearable devices, external noise). *Extensive* refers to the number of sensory modalities accommodated. *Surrounding* refers to the visual presentation of the VE, including field of view and the degree to which the physical world is shut out (e.g., head-mounted display, surround projection, computer screen). *Vivid* refers to the fidelity and resolution with which the VE simulates the desired environment (e.g., visual information, functionality). *Matching* refers to whether the viewpoint of the VE is modified to match the user's perspective through motion capture.

In this review, we classify VEs as low, moderate, or high immersion based on the extent to which they meet the criteria defined earlier (Table 1). When a study differed in level of immersion across multiple aspects, we averaged across criteria to determine a global immersion rating. For example, if an environment met low criteria on two aspects, moderate criteria on three aspects, and high criteria on one aspect, it was classified as moderate immersion.

A technology-based method of describing levels of immersion differs from a perspective-based method focused on the participant's subjective experience of the VE. Presently, there is insufficient qualitative or quantitative data on the participants' perceptual experiences to draw perspective-based conclusions, since few studies have measured this construct.^17^

### Sense of presence

*Sense of presence*, or the perceptual experience of being in a VE, is a function of level of immersion mediated by the context of a task and the perceptual thresholds of the participant.^16,18^ Brown and Cairns^19^ asserted that “full immersion is presence,” highlighting the influence that manipulation of level of immersion may have on a user's experience. These two concepts are distinct, in that immersion refers to a set of technical manipulations, while presence refers to a user's individual experience. However, since the level of immersion facilitates sense of presence, which in turn moderates learning effects, it is impossible to completely dissociate these constructs.

Sense of presence is closely tied to the visual and interactive fidelity of the VE,^20^ or how closely the VE matches the real world in appearance and functionality. Brown and Cairns^19^ suggested that *engagement*, or the ability to physically interact with and control a VE, is a core component of immersion, given its relationship to functional fidelity.^19,21–24^ However, engagement is presently best understood as a component of presence, and its relationship to immersion is not well established in the literature.

Individual differences and how readily individuals become immersed in the VE may also drive a sense of presence.^18^ The complex relationship between one particular component of immersion—ability to control and interact with the VE—and a user's resulting sense of presence also impacts effectiveness of learning.^21,25–27^ These findings from the typical development literature have implications for the use of VEs as a platform for treatment in ASD, since it is possible that some individuals with ASD may not achieve the sense of presence necessary to benefit from a VE-based intervention.

With notable exceptions,^17^ few studies of ASD have directly measured sense of presence and its potential influence on social skills training in a VE. Fewer still measured individual differences in subjects' tendency to become immersed in the VE,^28^ and to our knowledge, none has directly compared differences in the degree and type of engagement in the VE. Thus, our discussion is centered on level of immersion as a technical manipulation, and with the data currently available, we can only draw inference about the influence of this manipulation on the user's sense of presence in ASD.

We acknowledge that empirical testing is needed to establish whether manipulating aspects of immersion affect the perceptual experiences of people with ASD and typically developing individuals in similar ways. Therefore, the present discussion proposes a theoretical framework for quantifying and systematically testing hypotheses about the level of immersion as traditionally defined by Slater and Wilbur.^16^ Our aim in doing so is to promote a unified vocabulary and system of quantifying the level of immersion for the field, which in turn may facilitate future efforts to examine the role of this variable in the success of virtual interventions.

## Search Criteria

We conducted a systematic literature search using PubMed, Scopus, and Ebsco (including Academic Search Complete, Medline, PsycARTICLES, PsycINFO, HealthSource, Science and Technology Collection, and Psychology and Behavioral Sciences Collection). We limited our search to English language articles published in peer-reviewed journals before July 2015 using the following terms: autism, Asperger, ASD, virtual reality, VR, virtual environment, VE, virtual.

Two independent reviewers screened 178 abstracts for relevance and reached consensus on the resulting set of articles. Excluded articles were case studies,^29^ those which only included typically developing subjects or subjects broadly defined as having “special needs,”^30^ those which appeared in search results, but did not relate to the topic,^31^ or those which explored nonsocial navigation and action in VEs.^32–40^ Commentaries, letters to the editor, and brief opinion articles were also excluded. This resulted in 40 empirical articles, 12 reviews, theoretical or experimental design papers,^41,42^ and meta-analyses.

When only considering articles that specifically focused on social skills assessment and intervention in ASD, our search yielded 29 relevant publications reporting original data. These publications are presented in Table 2, classified by the social skill domain and level of immersion.

**Table 2.** Studies Using Virtual Environments to Assess or Teach Social Skills

*Author(s)*	*Year*	*Age*	*Sample*	*Primary purpose and VE task*	*Result*	*Level of immersion*
***Identifying emotions or intentions***						
Moore et al.	2005	7–16	ASD = 34	***Assess*** ability to make emotional judgments and causal inferences from virtual facial expressions	No difficulty accurately recognizing and inferring the source of characters' emotions	Low
Schwartz et al.	2010	20–53	ASD = 20 Control = 20	***Assess*** ability to interpret a virtual character's socially relevant/irrelevant movement and ***assess*** engagement	Less influence of socially meaningful gaze and facial expression in ASD group; lower sense of contact and urge to contact in ASD group	Low
Bekele et al.	2013; 2014	13–17	ASD = 10 Control = 10	***Assess*** ability to label emotions from animated facial expressions lip-synced to audio presentation of stories	No group differences in emotion recognition; less gaze toward mouth and eyes in ASD group	Low
Grynszpan et al.	2012	13–31	ASD = 14 Control = 14	***Assess*** ability to answer questions about emotion and intent from watching characters tell stories with related facial expressions and backgrounds	Atypical self-monitoring of eye movements, difficulty maintaining gaze, correlation between accuracy and fixation duration for ASD group	Low
Wallace et al.	2010	12–16	ASD = 10 Control = 14	***Assess*** sense of presence and ratings of the social attractiveness of a virtual character in a socially desirable/undesirable scenario using surround projection	No group difference in sense of presence; effect of desirable or undesirable scenario only present for TD, ASD group rated characters equally	Moderate
Cheng et al.	2010	8–10	ASD = 3	***Teach*** empathic accuracy using a self-expressive avatar and animated scenarios	***Improvement*** in empathic response for one subject; compliance issues for two subjects	Moderate
Kandalaft et al.	2013	18–26	ASD = 8	***Teach*** multiple social skills using a self-expressive avatar to interact with a virtual therapist in multiple contexts	***Improvement*** in ability to label and infer emotions, but not in conversational skills	Moderate
Kim et al.	2014	8–16	ASD = 23 Control = 23	***Assess*** ability to recognize emotions and choose to approach or avoid a virtual character using an avatar	Less approach to positive emotions for ASD group, equal avoidance to negative emotions	Moderate
***Conversation***						
Cheng and Ye	2010	7–8	ASD = 3	***Teach*** ability to interpret and respond to social behaviors using a self-expressive avatar to interact with virtual characters	***Improvement*** in Theory of Mind scores and socially appropriate behaviors	Moderate
Trepagnier et al.	2005; 2011	16–30	ASD = 16	***Teach*** conversation skills using verbal and nonverbal feedback from virtual actor and therapist	***No improvement*** in conversational skills; self-reports of VE being low stress and useful	Moderate
Strickland, Coles, and Southern	2013	16–19	ASD = 22	***Teach*** conversation skills using a virtual job interview with role play practice sessions, didactics, and feedback	***Improvement*** in content included in interview responses for intervention, but not the control group	Moderate
Smith et al.	2014	18–31	ASD = 26	***Teach*** conversation skills using a virtual job interview with role play practice sessions, didactics, and feedback	***Improvement*** in job interview performance for the intervention group relative to treatment-as-usual	Moderate
***Gesturing***						
Cheng and Huang	2012	9–12	ASD = 3	***Teach*** gestures using a data glove to manipulate virtual objects and interact with a virtual character	***Improvement*** in four joint attention skills; different levels of spontaneous gesturing	High
Wang and Reid	2013	6–8	ASD = 4	***Teach*** gestures using a virtual self-representation and hand gestures to move virtual objects	***Improvement*** in gesturing from baseline to postintervention	High
***Socially appropriate behaviors***						
Parsons et al.	2004; 2005	13–18	ASD = 12 Control = 12	***Assess*** adherence to social conventions when choosing whether to walk through a gap in a crowd in a virtual cafe	No group difference in adherence to social conventions; off-task behavior	Low
Rutten et al./Mitchell et al.	2003/2007	14–15	ASD = 7	***Teach*** socially appropriate behaviors through indirect feedback when choosing a seat in a virtual cafe or bus	***Improvement*** in socially appropriate behaviors after indirect feedback	Low
Kuriakose and Lahiri	2015	17–23	ASD = 2, Control = 3	***Assess*** behavioral and physiological responses (pulse rate, skin temperature, electrodermal activity) to avatar emotional displays that varied in valence and subtlety.	More anxiety-related physiological reactivity to avatar emotional displays for ASD group, but may be related to symptom severity.	Moderate
Lahiri et al.	2011	13–17	ASD = 6	***Teach*** appropriate gaze using a virtual classmate who tells a story and adjusts actions based on subject gaze	***Improvement*** in gaze appropriateness across trials; VIGART system usability validated	Moderate
Lahiri et al.	2013; 2015	13–18	ASD = 8	***Teach*** appropriate gaze and conversation skills using virtual characters telling stories with related backgrounds	***Improvement*** in gaze appropriateness and conversational skills across trials	Moderate
Bernardini, Porayska-Pomsta, and Smith	2014	5–7	ASD = 42	***Teach*** appropriate use of gaze and conversation skills using virtual characters and fixed response options	***Improvement*** in the appropriate use of opportunities to communicate with human and virtual interaction partners after intervention	Moderate
Jarrold et al.	2013	8–16	ASD = 37 Control = 54	***Assess*** ability to use gaze appropriately and answer self-referencing questions using a virtual classroom and peers	Impaired social attention in ASD group relative to controls, most pronounced for individuals with ASD and anxiety/attention symptoms	High
Maskey et al.	2014	7–13	ASD = 9	***Teach*** appropriate response to anxiety-producing scenarios or phobias using individualized VEs and tasks	***Improvement*** in response to anxiety/phobia in the real world 12–16 mos. postintervention	High
***Cooperation***						
Alcorn et al.	2011	5–14	ASD = 32	***Assess*** ability to cooperate with a virtual character and respond to feedback when picking virtual flowers	High task accuracy, lower RT in gaze-plus-gesture condition than gaze-only	Moderate
Stichter et al.	2014	11–14	ASD = 11	***Teach*** cooperative problem solving using the task of building a virtual restaurant with other virtual characters	***No improvement*** in social skills after iSocial intervention	Moderate

Studies labeled ***Teach*** also include an ***Assess*** component. Results labeled ***Improvement*** are considered successful implementations of a VE as a vehicle for intervention.

ASD, autism spectrum disorder; RT, reaction time.

## Assessing and Teaching Social Skills in VEs

Social skills vary in difficulty, with some acting as “building blocks” for more complex interaction. These skills, such as emotion identification, impact the ability to successfully perform more complex social behaviors, such as responding appropriately to the actions or emotions of others^43^ and cooperating to solve problems.^44^

Facilitating interaction with a VE by manipulating aspects of immersion may provide the additional context or scaffolding needed to help individuals with ASD successfully learn and demonstrate higher order social skills.^45^ Because a person is unable to physically touch elements of a VE, achieving a sense of presence in this context requires suspended disbelief, which in turn requires abstract thinking and imagination. These are areas in which individuals with ASD sometimes struggle. Therefore, increasing the level of immersion by making a VE more inclusive, extensive, surrounding, vivid, and matching (e.g., including multisensory feedback and modifying the VE to match the participant's perspective) reduces the degree of abstraction required.

More complex social skills (e.g., unstructured conversation) may require more scaffolding than simple skills such as emotion recognition. An individual with ASD may receive sufficient benefit from a low-immersion VE when learning to identify emotions from facial expressions, because this skill is largely visual and does not require a complex response. However, a high-immersion environment may be more effective for teaching conversation skills, which require integration of emotion and intention identification, gesturing, and receptive language.

### Identifying emotions or intentions

VEs have been used to assess differences in the ability of individuals with ASD to recognize and respond to emotions or intentions.^17,28,46–49^ Low-immersion VEs may not have sufficient sensitivity to detect performance differences on emotion recognition tasks^46,47,50^ (Table 2, section 1), despite clear differences in how people with ASD use gaze to obtain emotion-relevant information^46–48^ (Table 2, section 1). However, moderate-immersion VEs revealed subtle differences in how individuals with ASD responded to the emotions of virtual characters, despite demonstrating performance equal to controls. Individuals with ASD and typically developing controls were further differentiated in their manner of responding when studies used physiological measures in addition to behavioral observations. VEs are also an effective platform for teaching emotion and intention recognition.^51,52^

Only moderate-immersion VEs have been used in intervention studies and have effectively produced improvement in the ability of individuals with ASD to label, infer, and respond to emotions expressed by virtual characters.^51,52^ The utility of low-immersion VEs for teaching this social skill has not yet been evaluated.

### Conversation

Moderate-immersion VEs have been used to teach conversation skills in ASD, with mixed results (Table 2, section 2). There is a dearth of research on the use of VEs for assessment of conversation skills outside of an intervention study. Two moderate-immersion studies had an extremely small sample size^53^ or did not produce observable improvement in conversation skills, despite positive feedback from participants about the VE experience.^53,54^

In contrast, two studies with a slightly larger sample size produced significant improvement in conversation skills.^55,56^ Since both of these studies were in the domain of interview performance, it is somewhat difficult to draw conclusions about the broad utility of moderate-immersion VEs for teaching conversational skills in multiple contexts. Higher levels of immersion may be better suited to this skill, given that it is by nature a more interactive skill than emotion identification, for example. However, since no low- or high-immersion studies have been conducted on conversation, it is difficult to draw conclusions regarding the level of immersion.

### Gesturing

Only two studies have delivered social gesturing interventions to teach social gesturing in high-immersion VEs,^57,58^ and thus far, none has used VEs as an assessment tool to characterize gesturing differences in ASD compared to typical development. Improvements in participants' joint attention and spontaneous gesturing occurred across the course of both interventions (Table 2, section 3). However, small sample size and lack of randomization to treatment and control conditions limit the generalizability of these studies. As a result, it is difficult to draw broad conclusions about the utility and effectiveness of VEs and the impact of a different level of immersion on assessing and teaching socially appropriate use of gestures from the current literature.

### Socially appropriate behaviors

A number of intervention studies have demonstrated the utility of low-, moderate-, and high-immersion VEs as a tool for teaching socially appropriate behaviors.^59–65^ Current evidence most strongly supports the use of moderate- and high-immersion VEs to teach socially appropriate behaviors^59–63^ (Table 2, section 4).

Jarrold et al.^66^ successfully differentiated individuals with ASD from typically developing controls in their social attention and ability to answer self-referencing questions in a high-immersion VE. While Parsons et al.^67,68^ did not find group differences in adherence to social conventions in their low-immersion VE, they note that high rates of off-task behavior may have impacted their results. Thus, it is possible that low-immersion VEs are equally appropriate for use in assessing socially appropriate behaviors.

Most recently, Kuriakose and Lahiri^69^ used a moderate-immersion VE to assess the responses of two adolescents with ASD and three typically developing controls to emotions of varying valence and subtlety displayed by avatars. They used a combination of behavioral responses to questions and physiological measurements of arousal and anxiety (e.g., skin conductance and temperature, pulse rate). Participants with ASD exhibited more anxiety-related physiological activity in response to avatars' emotional displays. However, the purpose of this study was largely to demonstrate feasibility of this combined physiological and behavioral measurement system, and therefore, the authors present these data as preliminary.

When considered in combination, the results of studies on socially appropriate behaviors suggest that VEs are an appropriate tool for assessing and teaching socially appropriate behaviors in ASD. Current evidence is mixed regarding the use of low-immersion VEs for assessment of socially appropriate behaviors, with some studies able to detect differences between typical development and ASD^66^ and others finding no group differences.^67,68^ To date, only two studies have specifically assessed this skill in a nonintervention context. As more studies are conducted in this area, the accumulating evidence may provide stronger support for or against the preliminary conclusions we have drawn here.

Maskey et al.^63^ notably demonstrate the rich flexibility of high-immersion VEs and their utility for delivering individualized enduring treatments that promote skill transfer to real-world situations. Given that ASD is characterized by a great deal of heterogeneity in symptoms and functional impairments, this is an important contribution to intervention research. Although a 6-week intervention is not sufficient to track long-term skill transfer to the real world,^63^ the extant body of work in this area presents preliminary evidence of the effectiveness of VEs in teaching socially appropriate behaviors.

More importantly, these results suggest that social skills interventions delivered through VEs may produce an increase in real-world instances of spontaneous, successful social interaction. Longitudinal studies should be conducted in the future, specifically those that track participants' postintervention behaviors across a longer time period. This would aid in determining whether skill acquisition and improvement in VEs endure beyond the intervention period.

### Cooperation

Few studies have examined cooperation in ASD using VEs, or indeed in general, compared to other social skills such as emotion recognition. One study supports the use of moderate-immersion VEs for assessment of cooperative skills in ASD.^70^ VEs may, however, prove an effective platform for learning and practicing cooperative strategies by interaction with avatars or computer-generated characters. However, the only intervention study in this domain to date did not observe improved cooperation after attempting to teach this skill in a moderate-immersion VE.^71^ More work is needed in this area to determine whether VEs are an effective modality for teaching cooperation, and if so, what group characteristics, problem types, and environments are most appropriate for use in this type of setting.

## Discussion

Using aspects of immersion established in the typical development literature (Table 1), we propose a theoretical framework for assessing the global level of immersion used in future studies. This approach to analysis of immersion must be empirically tested to establish its validity and utility in studies of both typical and atypical development. In previous years, cross-study comparisons have presented significant challenges because of differences in methodology and terminology. Adhering to a unified definition of immersion and its components will facilitate this type of comparison across studies, enabling researchers to test the construct validity of a global immersion score. Use of this classification framework may also help researchers design studies that more carefully parse the impact of each aspect of immersion on the success of virtual interventions in ASD.

Despite the dearth of literature on the use of VEs in social skills interventions for ASD and the challenges that remain, important early conclusions about the role of immersion can be drawn from the studies reviewed here.

### Highly inclusive and surrounding VEs facilitate learning

One element determining the level of immersion is inclusion (Table 1). Studies of typical development suggest that more inclusive and surrounding VEs produce greater task engagement and motivation.^72,73^ Given the facilitative role of intrinsic motivation in learning,^74^ it follows that highly inclusive surrounding VEs may be optimal for improving social skills.

As we have reviewed here, both high- and low-inclusion VEs have been used in studies of social skills in ASD. In low-inclusion designs, the subject often has limited interaction with scenes or objects in the VE.^46,47,50^ These designs have been used to model social skills and to assess emotion recognition. Some researchers used full-body motion capture to enable participants to interact physically with objects in the virtual world, creating highly inclusive extensive VEs with viewpoints modified in real time to match the user's position.^75,76^ Others presented VEs on a desktop computer monitor and instructed participants to use a keyboard, joystick, or mouse to activate options (e.g., choosing the next question in a conversation) or interact with virtual objects.^64,65,68^ However, systematic research has not yet assessed the role of inclusion in the effectiveness of VE-based intervention.

Studies of typical development demonstrate an advantage of high inclusion when subjects participate in learning and memory tasks.^21,25–27^ However, highly inclusive VEs may sometimes be accompanied by a high degree of novelty. This may place additional strain on attentional resources, which in turn reduces the availability of cognitive resources for performing the target task.^77^ It is critical to understand the interplay between inclusion, attention, and motivation in studies that use VEs to deliver interventions, to optimize the learning environment.

Based on current literature in both the ASD and typically developing populations, we posit that context may determine the level of inclusion needed to effectively deliver VE-based intervention in ASD and that complex social skills may require greater inclusion to produce treatment effects. However, given the relationship between novelty and high inclusion found in the typical development literature, individuals with ASD may require additional practice sessions to habituate to the novelty of the VE before they are able to fully attend to the target task. Future studies of inclusion would benefit from comparison of groups who do and do not receive additional practice sessions to fully assess the role of novelty in the use of highly inclusive VE interventions to individuals with ASD.

### Level of immersion required to produce a treatment effect differs across individuals

We advocate for a careful analysis of the degree to which level of immersion impacts treatment response and the ability to generalize skills to the real world. At present, too much variability exists in the delivery method of VE-based interventions and assessments, and the level of immersion is not measured consistently. The criteria proposed by Slater and Wilbur^16^ may be a useful means of standardizing classification of level of immersion. Using this proposed classification system, our review identified 12 studies with either a moderate or high level of immersion (Table 2). Only two studies used low-immersion VEs to deliver interventions, perhaps because of practical barriers to teaching complex social skills in low-immersion environments. The two studies, which both reported improvements with the use of low-immersion VEs, are from the same group and only addressed the “socially appropriate behaviors” skill domain.^64,65^

In contrast, studies using moderate-immersion VEs were used to teach a variety of social skills such as identifying emotions or intentions, conversation, cooperation, and socially appropriate behaviors. Seventy-five percent of these moderate-immersion studies reported a positive treatment effect. Two studies also used high-immersion VEs to teach skills in the domains of gesturing and socially appropriate behavior, and both reported a positive treatment effect.

These results suggest that VEs with a higher level of immersion are more conducive to successful delivery of social skills interventions in ASD. However, more studies are needed to tease out the influence that each component of immersion exerts on treatment effects. Specifically regarding extensity, future studies should examine whether treatment response differs between traditional real-world settings, desktop computer VEs, large-scale projection-based VEs, and mobile devices. If effects are similar across modalities when holding all other aspects of immersion constant, desktop computer VEs and mobile devices may be viable options for populations with limited access to care.

VE intervention studies in ASD traditionally focused on assessment, learning, practice, and generalization. However, as we demonstrate in this review, few studies directly assess all four domains. Given the resources required to implement a VE intervention, it is necessary to demonstrate that any observed effects are (a) lasting, (b) flexible across contexts, and (c) consistent across individuals at a given level of functioning.

At present, many studies have assessed skills in a limited time frame and have not taken steps to determine whether treatment effects last longer than a few days or weeks postintervention. Even fewer have tested the impact of skills learned in the VE on real-world functioning. Interventions should be pursued beyond the laboratory to assess the impact of level of immersion on the transfer of skills to the real world, and attention should be given to whether those skills generalize to multiple contexts. To achieve this goal, tasks delivered through an immersive VE must be ecologically valid, with environments and characters that have high visual and interactive fidelity, close matching between the VE and the user's perceptual experience, and extensive multisensory feedback. Future research is needed to determine sensory thresholds for specific tasks in the domains of extensity, vividness, and matching. Like inclusion, these components of immersion may differ in the amount of influence they have on treatment effectiveness dependent upon the specific context and target skill. This will ensure that the VE is optimized for learning and skill transfer, as previously discussed. This includes consideration of cultural validity, which differs across communities, educational settings, and socioeconomic backgrounds.

Finally, when comparing the positive user experiences reported by Trepagnier et al.,^53,54^ Strickland et al.,^55^ and Smith et al.^56^ to the difficulties reported by Cheng et al.^51^ in their studies of conversation skills, we clearly see the importance of screening participants for sufficient ability to perform the tasks administered in the VE. To determine who will benefit most from VEs, and who will be better suited to traditional intervention formats, researchers will need to selectively recruit samples that are controlled to minimize developmental effects and differences in symptom profiles.

Future studies must also determine visual and interactive fidelity thresholds for individuals with ASD. Understanding these thresholds may aid researchers in facilitating a more immersive experience. By virtue of the technology required to achieve high extensity and matching, highly immersive environments may offer more opportunities for engagement with the VE, in turn producing a stronger sense of presence.^72,73^ Studies of typical development suggest that high-engagement conditions are optimal for learning in the VE.^21,25–27^

It may be the case that highly immersive VEs are also optimal for individuals with ASD or, conversely, that specific phenotypes on the autism spectrum respond differently to different levels of immersion. For individuals with extreme sensory sensitivities, highly extensive and vivid VEs may prove overwhelming. Alternately, for sensory-seeking individuals, these types of VEs may prove distracting.

Few studies of ASD assess the ability or desire to be immersed in a VE.^17,30^ Individual differences in these domains may help to separate those who are likely to benefit from VE-based intervention from those who require face-to-face settings and those who learn effectively in low-immersion VEs from those who require higher visual and interactive fidelity and higher levels of immersion.

The conflicting findings of Wallace et al.^17^ and Schwartz et al.^28^ suggest that the ability or desire to interact with a VE may differ across contexts or between individuals with ASD. Atypical comprehension of pretense and difficulty pretending has been documented in ASD,^78–83^ and either of these clinical features may impact an individual's tendency or capability to be immersed in a VE. Therefore, it is not only the visual and interactive fidelity of the VE that drives the perceptual experience in this population. Ability and willingness to interact with and be immersed in a virtual world may be altered in some individuals with ASD, and if so, VE-based intervention may not be an appropriate format.^28^ In addition to studying the impact of level of immersion on VE interventions, we must also understand what makes someone a good candidate for this platform. The available body of work does not directly address these remaining questions, so future studies may benefit from obtaining detailed symptom profiles and examining subgroups within a broader sample of individuals with ASD.

## Conclusions

Low-immersion VEs are sufficient to detect some differences in social performance.^28,70^ Exceptions^67,68^ may be due to participant characteristics, including tendency to be immersed, attention, and symptom severity. However, the literature on intervention studies is considerably less straightforward. In one instance, low-immersion VEs produced improvement in social skills.^64,65^ However, while some moderate-immersion VEs have produced improvements,^60–62^ others have not.^53,54,71^

Notably, these conflicting results occur in different skill domains—socially appropriate behavior and gaze versus cooperation and conversation—which may explain the variable effectiveness of moderate immersion. Thus, it is important to understand the role of task complexity independent of the level of immersion. For high-immersion VEs, treatment response was overwhelmingly positive.^52,55,56^

The current body of work suggests that VEs may offer an appropriate avenue for delivery of social skills therapies for some individuals with ASD. The potential advantage to using VEs in place of more traditional measures or intervention approaches lies in the ability to generate more ecologically valid tasks and to teach and assess skills under conditions that more closely mimic the real world. Additional research is needed to determine whether this approach is equivalent to traditional face-to-face intervention.

Larger sample sizes and randomized controlled trials would help to illuminate what differences, if any, exist between these two formats. Most research using VEs in social skills applications is conducted in a pre- and post-test design without random assignment or an assessment of skills without intervention. Thus, it is currently easier to draw conclusions about the utility of this platform for the assessment and practice of existing social skills than as a medium for teaching new skills.

Several characteristics of VEs determine level of immersion, which in turn facilitates the sense of presence and learning. Considering six aspects of immersion, we proposed a theoretical framework for systematical classification and reporting of level of immersion in future studies using VEs. Our hope is that through subsequent testing and refinement of the proposed theoretical framework in Table 1, researchers will gain a better understanding of the influence of immersion on the effectiveness of VEs for assessment and treatment of the social symptoms of ASD.
